# Highlights from the Student Council Symposium 2011 at the International Conference on Intelligent Systems for Molecular Biology and European Conference on Computational Biology

**DOI:** 10.1186/1471-2105-12-S11-A1

**Published:** 2011-11-21

**Authors:** Priscila Grynberg, Thomas Abeel, Pedro Lopes, Geoff Macintyre, Lorena Pantano Rubiño

**Affiliations:** 1 Departamento de Biquímica e Imunologia, Instituto de Ciências Biológicas, Universidade Federal de Minas Gerais, Belo Horizonte, Minas Gerais, Brazil; 2 VIB Department of Plant Systems Biology, Ghent University, Technologiepark 927, 9052 Zwijnaarde, Belgium; 3 Broad Institute of MIT and Harvard, 7 Cambridge Center, Cambridge, MA 02142, USA; 4 DETI/IEETA, University of Aveiro, Campus Universitário de Santiago, 3810 – 193 Aveiro, Portugal; 5 NICTA Victoria Research Laboratory, The University of Melbourne, Melbourne, Victoria 3010, Australia; 6 Department of Regulatory Genomics, Institut de Medicina Predictivai Personalitzada del Càncer, Badalona, Catalonia, Spain

## Abstract

The Student Council (SC) of the International Society for Computational Biology (ISCB) organized their annual symposium in conjunction with the Intelligent Systems for Molecular Biology (ISMB) conference.

This meeting report summarizes the scientific content of the Student Council Symposium 2011 as well as other activities organized by the Student Council in the context of ISMB. The symposium was held in Vienna, Austria on July 15^th^ 2011.

## Introduction

The ISCB Student Council is a worldwide organization for students in bioinformatics and computational biology. Its main goals are the organization of events and the creation of networking opportunities for students. The main contribution of the SC is to nurture students' soft skills, such as teamwork and group dynamics, and leadership and cooperation skills, to improve their regular academic program. Since its inception, the Student Council has organized an annual student symposium for the benefit of the student community. This year, the Student Council Symposium was held in conjunction with the ISMB/ECCB conferences on July 15th. Over 85 delegates attended this edition of the Student Council Symposium. The event opened with a well-received scientific speed dating session, allowing the delegates to get to know each other before the sessions kicked off. The program featured three keynote lectures, two partner presentations, ten student oral presentations and an extensive poster session with over 50 posters.

We were honored to have three highly esteemed scientists deliver the keynote presentations. Dr. Chad Myers (University of Minnesota, Minneapolis, MN, USA) opened the scientific program with a presentation entitled: “Systems-Level Insights from Large-Scale Combinatorial Perturbation Experiments in Yeast”. The afternoon session started with Dr. Ivet Bahar (University of Pittsburgh, Pittsburgh, PA, USA) presenting “Protein Dynamics: Learning from Computations and Experiments”. The closing keynote lecture of the day was conducted by Dr. Curtis Huttenhower (Harvard School of Public Health, Boston, MA, USA) and was titled “Functional Metagenomics and the Human Microbiome”.

This year we welcomed two talks by our industry and research partners. Dr. Izhak Haviv of Australia`s Information and Communications Technology Research Center of Excellence (NICTA) and the Department of Pathology, University of Melbourne (Melbourne, Australia) gave an overview of the new efforts to reduce the processing time of algorithms used for genomic data analysis. Janna Hastings of the European Bioinformatics Institute (Cambridge, UK) provided insight into training and career opportunities for students and other early career scientists at EMBL-EBI.

During the symposium, the audience was treated to a presentation from three students taking part in the Student Council Internship Program (http://www.iscbsc.org/internships). This new initiative from the Student Council has resulted in four students from developing nations participating in internships of three to six months at two research laboratories in Europe, hosted by Dr. Burkhard Rost and Dr. Reinhard Schneider. The three internship students, Maina Bitar from Brazil, Mohd Rehan from India and Dedan Kinuthia Githae from Kenya, provided symposium attendees a snapshot of their experiences during their time as interns and the benefits of participating in the program.

## Proceedings

This year we received almost one hundred submissions from students to present their work at the symposium. These submissions were peer-reviewed by 39 independent reviewers from whom the program committee selected 10 for oral presentation, of which 9 are included in this meeting report. An additional 50 abstracts were accepted for poster presentations and the three winners of the best poster awards also got the opportunity to have their abstract included in this report. The nine included oral presentations fall into three main fields of research: Sequence analysis, Bioinformatics tools, and Transcriptomics: gene expression analysis and data management. Each of these topics features a block of three or four presentations. Below is a short discussion of the presentations that are featured in the special proceedings issue.

### 1. Sequence analysis

The DNA, RNA and protein sequences generated from thousands of organisms and stored in public databases has facilitated the development of several research fields, including genome assembly and annotation, sequence alignment and polymorphism search, and protein structure prediction. Comparison of sequences provides interesting insights about the relationship between genotype and phenotype. A good example of this was presented during the symposium by Klotzbuecher* et al.*[1]. They showed a new method for determining which pairs of single nucleotide polymorphisms (SNPs) affect the flowering time of *Arabidopsis thaliana*. The method consisted of a two-step approach to determine pairs of interacting SNPs that are strongly associated with the phenotype. The first step used a branch-and-bound strategy to prune away insignificant pairs. The second step applied prior biological knowledge to reduce the search space.

Recently, the advent of new sequencing technologies has altered the bioinformatics research landscape considerably, pushing the boundaries of what is considered high-throughput. Researchers are now able to sequence complete genomes, transcriptomes and exomes in a fast and cheap way. The large amount of data generated by such projects mean that new approaches and methods of analysis are required. As an example, Buske* et al.*[2] presented methods for analyzing sequence data from the exome of 1,000 individuals with autism in an attempt to discover associated genetic variants. The development of a specific analysis pipeline and tools for alignment, variant detection, and data visualization was demonstrated.

The advent of large scale sequencing technologies has also impacted evolutionary studies. Instead of a focus on single genes, or small groups of genes, it is now possible to sequence complete genomes from several samples or species, and study evolution from a global perspective. McElroy *et al.*[3] deep-sequenced the genome of *Pseudomonas aeruginosa* PAO1, a biofilm model of lung infection. Results showed that the bacteriophage, and not the bacterial genome, was undergoing diversification. To help with the analysis, the authors developed cutting-edge statistical techniques to reconstruct bacteriophage haplotypes.

### 2. Bioinformatics tools

Software tools and databases are becoming increasingly important for the analysis, visualization and comprehension of the molecular data. In this session, different topics were covered from genomic analysis to protein analysis.

Genome-wide association studies (GWAS) are a powerful approach to determine the association between variations from different individuals from the same species with specific phenotypes. GWAS analyses encounter limitations as a result of large datasets generated. Goudey *et al*. [4] presented a novel algorithm known as Optimal Pairwise Epistasis (OPE) for exhaustively examination of all pair-wise SNP interactions in GWAS. The robustness of the tool was validated through analysis of two independent datasets from Celiac patients. They also showed a 10-fold decrease in time consumed for the analysis, compared to other approaches.

A significant contribution to biological research has been the development of *in silico* prediction tools. They allow many experiments to be conducted initially *in silico*, saving time and money. Woodcroft *et al*. [5] demonstrated the development of an integrative bioinformatics predictor of protein sub-cellular localization in malaria. More than 700 publications stating the sub-cellular location of a protein were used to train and test the method. The method achieved an accuracy of approximately 60% on a seven class problem. To validate the *in silico* results, the localization of a number of proteins was verified experimentally.

Recent sequencing technologies require the development of new software suites to analyze the large amount of data generated in a quick and user-friendly way. Schultheiss *et al*. [6] showed a powerful and effective framework to analyze RNA-seq data in less than 20 minutes with a machine-learning workflow integrated in an easy-to-use Galaxy framework. The modular tool suite deals with different aspects of a typical next-generation analysis: short-read alignments, transcript identification/quantification and differential expression analysis.

### 3. Transcriptomics: gene expression analysis and data management

Gene expression changes are one of the fastest and most versatile responses of an organism facing changes in their environment. The comprehension of this scenario is essential to understand an organism or cell adaptations and survival in response to stress.

Several techniques are available for measuring levels of gene expression, such as Expressed Sequence Tags (EST), Serial Analysis of Gene Expression (SAGE), microarrays, RNA-seq, and Cap Analysis of Gene Expression (CAGE). During the symposium, Franscescatto *et al.*[7] showed an application of some of these technologies. They used CAGE combined with high-throughput sequencing (deep-CAGE) to collect specific transcription start sites (TSS) and their expression levels in different areas of human aged brain. These were used to correlate expression with methylation and structural genomic variation.

The reliability of gene expression studies depends largely on the method of analysis, including solid statistical approaches. For tumor gene expression analysis studies, an extra issue must be considered: tumor samples, when extracted from solid cancers, also contain healthy tissues, and this can compromise the cancer gene expression pattern. A new computational method was developed by Deshwar *et al*. [8]to purify tumor gene expression profiles using reference samples of healthy tissue to model the real contribution of cancer samples.

Collections of comprehensive studies that generate large amounts of data usually result in two or more different sources of data that are complementary each other. Databases are important tools to store, organize and utilize such data. Bovolenta *et al*. [9] presented the Human Transcriptional Regulation Interactions database (HTRIdb), an open-access database of experimentally validated interactions among human transcription factors and their respective target genes.

### Poster award winners

This year, the SC awarded three poster presentations. Awards were decided by delegate voting. The third prize was awarded to Mrinal Mishra [10]. The authors analyzed the genetic network of five pancreatic cancer candidate genes using a Cytoscape plugin. They found that two important candidate genes interact with 21 neighbor genes, revealing the importance of a change in expression level of candidate genes in causing pancreatic cancer. The second prize was given to Emre Guney [11], who presented a new framework called GUILD (Genes Underlying Inheritance Linked Disorders). The idea was based on the application of methods to prioritize the relation between genes and disease to reveal pathways associated with the disorder. First place went to Benjamin Kwan [12] who presented a new numerical representation and novel thresholding technique for classifying short exon and intron sequences using discrete Fourier transform period-3 value.

## Student Council activities at ISMB

### 1. Student Council career activities

During ISMB the Student Council organized a Career Session designed to provide students with information to help them make informed decisions about their future careers. The first half of the session saw Dr. Jaap Heringa, Director of the Centre for Integrative Bioinformatics at Vrije Universiteit Amsterdam, The Netherlands, give a talk on his career choices and outline the important factors to consider when pursuing a career in bioinformatics. Following Jaap’s talk, a career discussion panel made up of five early career researchers, Dr. Nils Gehlenborg, Dr. Jeroen de Ridder, Dr. Yana Bromberg, Dr. Magali Michaut and Dr. Daniela Maisel along with Dr. Jaap Heringa took questions from the audience. The panelists gave valuable insights while answering interesting and at times provocative questions from the student audience.

Besides the Career Session in the conference program, the Student Council provided an enhanced job advertising process throughout the meeting in the exhibitors hall. Job advertisers could meet with job seekers through our interactive job posting board. As a result, many impromptu interviews were carried out at the Student Council booth during the conference with positive feedback from participants, both interviewers and interviewees.

### 2. Social activities

As networking opportunities are a major benefit of participating in a conference, the Student Council focused on organizing activities outside conference hours to continue discussions and transference of knowledge. The daily Social HQ saw up to 50 students meet up over dinner each night, providing the ability for students to network and discuss their scientific problems in more detail. The Student Council Social Event was also a highlight with approximately 70 students participating in a walking tour of Vienna followed by dinner and a quiz. The social events provide a fun and relaxed atmosphere for students to connect and discuss all things bioinformatics and more.

## Conclusions

The number of abstract submissions for the Student Council Symposium is growing every year. This year we were once again able to organize a program with three high-quality student presentation sessions, a broad poster session and three invited keynotes. Coupled with the SC activities organized during the main conference, the Student Council Symposium at ISMB/ECCB is fast becoming the premier event for students in bioinformatics and computational biology.

## The future

Next year, the Student Council will feature two major international events. First, the Student Council Symposium will be held together with ISMB 2012, in Long Beach, CA, USA. Second, The European Student Council Symposium will enrich ECCB 2012, in Basel, Switzerland. Further information regarding the Student Council, its events, internships and community, please visit http://www.iscbsc.org.
